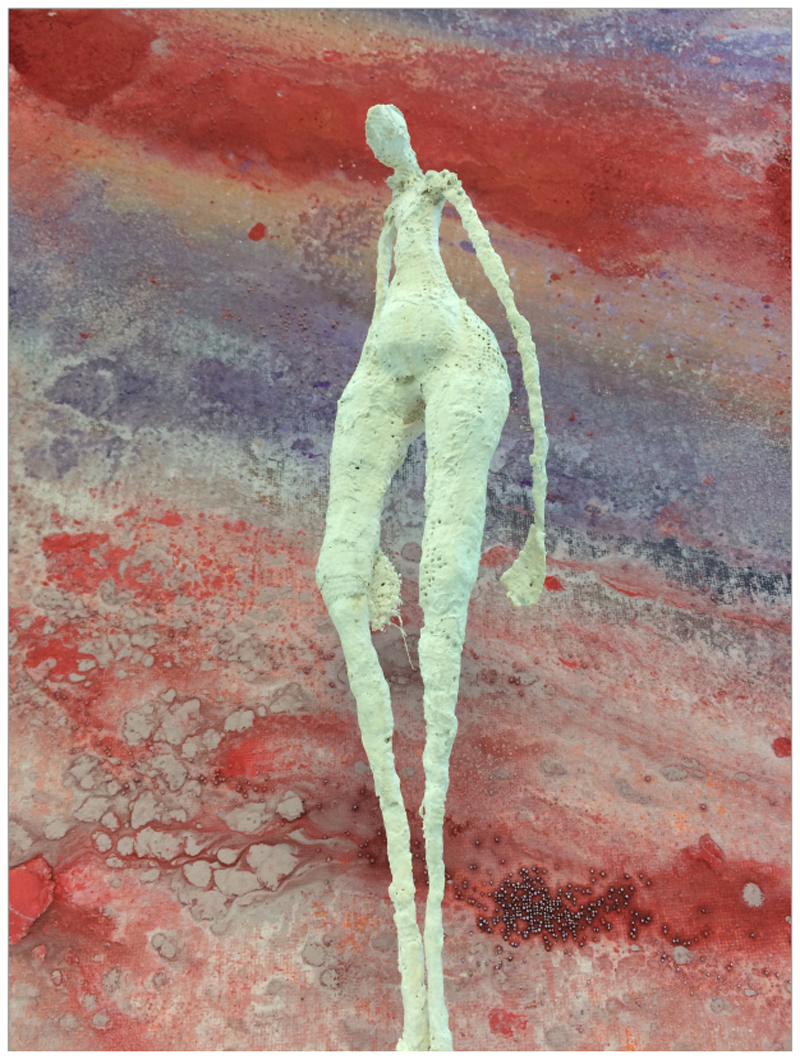# The art of medicine “A taste of your own medicine”: the experience of a transplant

**DOI:** 10.1016/S0140-6736(25)00691-9

**Published:** 2025-04-12

**Authors:** Havi Carel

**Affiliations:** Department of Philosophy, University of Bristol, Bristol BS6 6JL, UK

It’s done. You’ve had it.” My partner is grinning widely. I open my eyes but have no clue where I am. He tells me I had a double lung transplant and I am in the intensive care unit. At first, I am confused. Then in pain. Then frightened. I doze and wake, not sure if it is a new day, or whether it is daytime at all. I do not understand what is going on around me.

My glasses are in a box somewhere and it takes a few days for me to convey that I need them. In the meantime, I have double vision and everything is blurry. My fentanyl-addled brain marvels at the staff around me in the UK National Health Service (NHS): these synchronised pairs, wearing matching uniforms. Here are the twins that empty bins. Here are the twins that clean the floor. I hallucinate, trying to piece together my fractured reality. I stare for hours at what I take to be a truly alarming Halloween display (it is August) and take comfort from the small girl sitting next to me quietly reading a book. I am upset to find out my mother is lying in the bed next to me, having sustained a head injury on the way to visit me. I am stressed about the storm and our evacuation to the roof, which goes very smoothly indeed: I didn’t even get wet. I recall a nurse coming to me and saying “you’re taking too long. You’ve got 48 hours to get better or we’re going to stop treating you”. “Please”, I say, “please, what about my children?

I contemplate escape but realise I’m tethered to a ventilator, a central line, electrocardiogram machine, and more monitors than I can count. Lying down, I labour to work out what is happening, try to listen to the nurses’ whispered handover conversations. Gentle hands wash and clean me; attention is lavished on me: observations, blood tests, x-rays (those twins with the portable x-ray machine). I get turned over with daily (and more) bedding changes. I am baffled by how I am still alive, without eating or drinking. My burning thirst is indescribable.

This mess inside me cannot be articulated because I cannot speak. I have no voice because of the tracheostomy. I am given a laminated sheet with the alphabet printed on it. But my hands are too shaky and brain too foggy to use it. So I lie there trying to get some mental order back, despite the bodily chaos. I am shown a schematic diagram of my tracheostomy. I am perpetually terrified that the little balloon in my trachea will self-inflate leaving me to suffocate in silence. (Note to anaesthetists: please do not put a patient on a ventilator without warning and then say “don’t bother trying to breathe”. It is very disconcerting.)

One day, a physiotherapist comes with a small box. It is a voice—my voice. She inserts it into the tube connected to the tracheostomy. Suddenly I can speak and be heard. But they take it away after a few hours (I am still ventilated some of the time) and I am bereft. I once again cannot explain anything I think or want or need. I am voiceless not as metaphor but fact. I am barely an epistemic agent at all.

I want my old body back. Everything in my new body hurts or stings or does not work or all three. I am exhausted and uncomfortable and my wounds are infected and need to be reopened—vacuum pumps are added, as is a weekly visit to theatre for debriding.

Slowly—extremely slowly—I get better. A speech therapist approves me for eating and drinking. I must take a few sips of water and bite on a cookie. But I am still also fed via a tube dangling from my nose. I commit to memory and thank each of the nurses who take ‘plastic’ out of me—the central line, the feeding tube, the chest drains, the catheter, eventually the tracheostomy. After 3 weeks I am moved to a high dependency unit. I have my own room. With a window that opens. It is so quiet. The tree outside so green.

What feels so eventful to me is routine for the people caring for me. By that, I do not mean that they are callous. On the contrary, the kindness of the nurses astonishes me. But I am still frail and exhausted and overwhelmed by the major surgery, medication, and hospital environment. A needle prick is just a needle prick. A shot of iron cheerily squirted into your mouth is also just a small insult to your senses. So are the heat waves from the intravenous magnesium. A chest drain is not a big deal, nor is washing in tepid water. The bump of the wheelchair into the lift is only a small bump. But my body is hypersensitive and emaciated and the cumulative effect of this continuous assault on my senses causes me enormous distress. The sheets are itchy, the blankets too heavy, my skin burns, my eyes dry, everything tastes terrible, monitors and bells are constantly ringing, it is never properly dark. Everything is out of kilter.

I am given an antibiotic with the colour and texture of fresh paint. The taste is abominable to me in my distressed and hypersensitised state. Each time I take it, I gag. It is emblematic of the need for patients to be involved more in the planning of care and securing their agency somewhere in this sensory overload. I ask the nurses if they have ever tried any of the stuff they give the patients. No, they all say. I invite the reader to have a taste: a feast of potions and electrolytes.

The medical team is unrelenting: they can see the progress even if I can’t. They listen attentively to my repetitive, weepy questions. A nurse patiently holds my hand as I retch into a bowl. A young cardiologist quietly convinces me that things will get better. My 82-year-old mother, Cynthia, is with me every day for months on end. Those are moments of grace and compassion that carry me along.

What has happened to me? I call it radical bodily doubt. As a philosopher researching the experience of illness using phenomenology, I offered the concept of bodily doubt in 2013 to describe the breakdown of the tacit, taken for granted sense of certainty or trust in our body. This certainty is a habitual confidence in the reliability of one’s bodily workings and capacities. Bodily doubt ensues when bodily certainty breaks down, for example in illness. It consists of, first, a loss of continuity: one starts to doubt their body in a way they had not previously. Second, there is also a loss of transparency: the taken for granted familiar functioning of the body’s organs is replaced by hypervigilant attention to the effects of illness. And third, there is a loss of faith in one’s body: what was previously comfortably trusted, suddenly becomes a dangerous traitor.

Radical bodily doubt is conceptually and experientially more extreme. It is emblematic of liminal situations, such as those in the intensive care unit or at the end of life (although there is a broad spectrum of experiences), in which bodily doubt hardens into the certainty of incapacitation. What is encountered is not bodily doubt, but a certainty of bodily collapse: bodily abilities are gone, not distrusted. The person experiences a destruction—partial or complete—of their embodied agency. It is no longer a doubt (“will I be able to do this?”) but a radical certainty: (“I am completely broken and unable to do this”). What is experienced as fragile and precarious in bodily doubt is experienced as lost in radical bodily doubt.

In radical bodily doubt there is bodily chaos and the loss of the vantage point anchoring one in the world. The loss of continuity is now its complete breakdown. As a result, one’s bodily presentation collapses and what remains is a medicalised and a scientised body. As physician Matthew Broome, reflecting on a period working in an intensive care unit, put it to me, “subjectivity and patients almost drop away…it is sort of applied physiology”.

The loss of transparency becomes a complete barrier that stops all normal human exchange, a barrier with no corrective other than getting better. And finally, the loss of faith in one’s body is replaced by a new certainty: the negation of possibility.

This passivity is constituted by one’s physical state: ill people in intensive care units can do almost nothing for themselves. Their ability to advocate for themselves is compromised. But it is also constituted interpersonally by the health professionals who look after the incapacitated person, not through malice or any attempt to dominate but simply as a result of the medical setting. This passivity is baked into the interpersonal dynamics: the patients are mostly unconscious, disoriented, or unable to speak; there are real physical and communicative barriers. Other people enable or disable the ill person’s most basic agency. Bodily capacities are appropriated: someone feeds a patient through a nasogastric tube; another person decides if a patient can eat or drink. One risks being reduced to a passive, manipulable object before the clinical-other, not just for a moment but in an enduring way. Those who care for you play important roles in sustaining, restoring, or further eroding a sense of bodily subjectivity and agency. That, too, will need to be repaired, not only the critically ill body.

I have now had many months of interactions with caring, committed, smart, health professionals. But do they know the terror of this radical bodily doubt? After decades of bodily and agential autonomy when I had the transplant, things were done to me. Even if I understood both their necessity and urgency, I was diminished by the passivity I had no choice but to be complicit in.

I was, ironically, called for my transplant just as I started work on a new Wellcome-funded research project on epistemic injustice in health care. The project, in part, develops accounts of illness, to ameliorate their neglect, so they do not remain occluded: unspoken and unheard. In a health-care world of multiple perspectives, we all need to occasionally take up another’s point of view and have a taste of our own medicine.

## Figures and Tables

**Figure:**